# Fisetin, a 3,7,3′,4′-Tetrahydroxyflavone Inhibits the PI3K/Akt/mTOR and MAPK Pathways and Ameliorates Psoriasis Pathology in 2D and 3D Organotypic Human Inflammatory Skin Models

**DOI:** 10.3390/cells8091089

**Published:** 2019-09-15

**Authors:** Jean Christopher Chamcheu, Stephane Esnault, Vaqar M. Adhami, Andrea L. Noll, Sergette Banang-Mbeumi, Tithi Roy, Sitanshu S. Singh, Shile Huang, Konstantin G. Kousoulas, Hasan Mukhtar

**Affiliations:** 1 School of Basic Pharmaceutical and Toxicological Sciences, College of Pharmacy, University of Louisiana at Monroe, Monroe, LA 71209-0497, USA; sbanang@yahoo.fr (S.B.-M.); royt@warhawks.ulm.edu (T.R.); singhss@warhawks.ulm.edu (S.S.S.); 2 Division of Allergy, Pulmonary and Critical Care Medicine, Department of Medicine, School of Medicine and Public Health, Madison, WI 53706, USA; sesnault@medicine.wisc.edu (S.E.); alnoll@wisc.edu (A.L.N.); hmukhtar@dermatology.wisc.edu (H.M.); 3 Department of Dermatology, School of Medicine and Public Health, University of Wisconsin, Madison, WI 53706, USA; vadhami@dermatology.wisc.edu; 4 Department of Biochemistry and Molecular Biology, Louisiana State University Health Sciences Center, 1501 Kings Highway, Shreveport, LA 71130-3932, USA; shuan1@lsuhsc.edu; 5 Feist-Weiller Cancer Center, Louisiana State University Health Sciences Center, Shreveport, LA 71130-3932, USA; 6 Department of Pathobiological Sciences, School of Veterinary Medicine, Louisiana State University, Baton Rouge, LA 70803, USA; vtgusk@lsu.edu

**Keywords:** fisetin, psoriasis, normal human epidermal keratinocyte, cell signaling, cell differentiation, proliferation, inflammatory cytokine, PBMC, CD4+ T lymphocyte, 3D psoriasis-like skin disease model

## Abstract

Psoriasis is a chronic immune-mediated skin disease that involves the interaction of immune and skin cells, and is characterized by cytokine-driven epidermal hyperplasia, deviant differentiation, inflammation, and angiogenesis. Because the available treatments for psoriasis have significant limitations, dietary products are potential natural sources of therapeutic molecules, which can repair the molecular defects associated with psoriasis and could possibly be developed for its management. Fisetin (3,7,3′,4′-tetrahydroxyflavone), a phytochemical naturally found in pigmented fruits and vegetables, has demonstrated proapoptotic and antioxidant effects in several malignancies. This study utilized biochemical, cellular, pharmacological, and tissue engineering tools to characterize the effects of fisetin on normal human epidermal keratinocytes (NHEKs), peripheral blood mononuclear cells (PBMC), and CD4+ T lymphocytes in 2D and 3D psoriasis-like disease models. Fisetin treatment of NHEKs dose- and time-dependently induced differentiation and inhibited interleukin-22-induced proliferation, as well as activation of the PI3K/Akt/mTOR pathway. Fisetin treatment of TNF-α stimulated NHEKs also significantly inhibited the activation of p38 and JNK, but had enhanced effect on ERK1/2 (MAPK). In addition, fisetin treatment significantly decreased the secretion of Th1/Th-17 pro-inflammatory cytokines, particularly IFN-γ and IL-17A by 12-*O*-tetradecanolylphorbol 13-acetate (TPA)-stimulated NHEKs and anti-CD3/CD28-activated human PBMCs. Furthermore, we established the in vivo relevance of fisetin functions, using a 3D full-thickness human skin model of psoriasis (FTRHSP) that closely mimics in vivo human psoriatic skin lesions. Herein, fisetin significantly ameliorated psoriasis-like disease features, and decreased the production of IL-17 by CD4+ T lymphocytes co-cultured with FTRHSP. Collectively, our data identify the prodifferentiative, antiproliferative, and anti-inflammatory effects of fisetin, via modulation of the PI3K-Akt-mTOR and p38/JNK pathways and the production of cytokines in 2D and 3D human skin models of psoriasis. These results suggest that fisetin has a great potential to be developed as an effective and inexpensive agent for the treatment of psoriasis and other related inflammatory skin disorders.

## 1. Introduction

The physical and immunological protective skin barrier function, mainly executed by the outermost epidermis, is sustained through the tight regulation of epidermal keratinocyte proliferation and differentiation, ultimately resulting in a dense, impenetrable stratum corneum [1]. In the normal epidermis, keratins are the most abundant structural proteins that form the keratin intermediate filament cytoskeleton [2]. Upon commitment to terminal differentiation, keratinocytes switch from expressing proliferative keratin 5 and 14 (K5 and K14) in the basal epidermis to expressing the early differentiation keratin 1 and 10 (K1 and K10) and associated induction of various differentiation-related protein markers in the suprabasal epidermis, which upholds the skin barrier’s integrity. These markers include keratin tonofilaments, the filament aggregating protein (filaggrin) [1,3,4], caspase-14 [5], the enzyme transglutaminase [4], and loricrin, an impenetrable late cornified envelope protein. Increased interactions between these proteins cause beneficial effects: enhancement of the skin’s natural moisturizing factor and epidermal hydration; maintaining the stratum corneum barrier function and skin integrity; and protecting the induction of inflammation by external stimuli. This cognate process is dysregulated in several chronic inflammatory skin diseases including psoriasis and atopic dermatitis, where lesioned skin keratinocytes exhibit hyperproliferation and aberrant differentiation, two crucial disease hallmarks [6]. Moreover, the expression levels of several differentiation-related proteins including caspase-14 [7], filaggrin [7], and loricrin [4,8] are downregulated in inflamed, porokeratotic, psoriatic skin lesions compared to nonlesioned and normal skin. Skin keratinocytes serve as the principal source of several pro-inflammatory mediators, including the cytokines interleukin (IL)-1, IL-6, IL-8, IL-10, IL-12, IL-15, IL-18, and IL-20, tumor necrosis factor (TNF) [8,9], and the chemokines CXCL8, CXCL11, and CCL20 [10]. In response to local stimuli, the production of these mediators can initiate an inflammatory process in psoriasis. In addition to keratinocytes, immune cells play a predominant role in chronic inflammatory skin diseases via the production of mediators such as IFN-γ, IL-17, IL-22, IL-23, IL-36, and the chemokine IP-10 [11,12,13,14,15,16,17]. Keratinocytes also possess cytokine receptors that serve as targets for activated T lymphocyte-derived IL-17 and IL-22, resulting in increased proliferation, aberrant differentiation, and further cytokine production by keratinocytes [8].

Agents that possess direct antiproliferative, prodifferentiative, and anti-inflammatory effects in epidermal keratinocytes and immune cells are potentially ideal candidates for treating inflammatory skin disorders such as psoriasis. Efforts to develop treatments for psoriasis remain elusive, mostly relying on small molecules and biologics [11,12,13,14]. Most of these treatments have significant limitations, including high cost, the need for injections, adverse drug reactions, and a loss of efficacy over time [15]. Thus, there is a need for the discovery and development of new, safe, and effective mechanism-based therapeutics.

**Figure 1:**
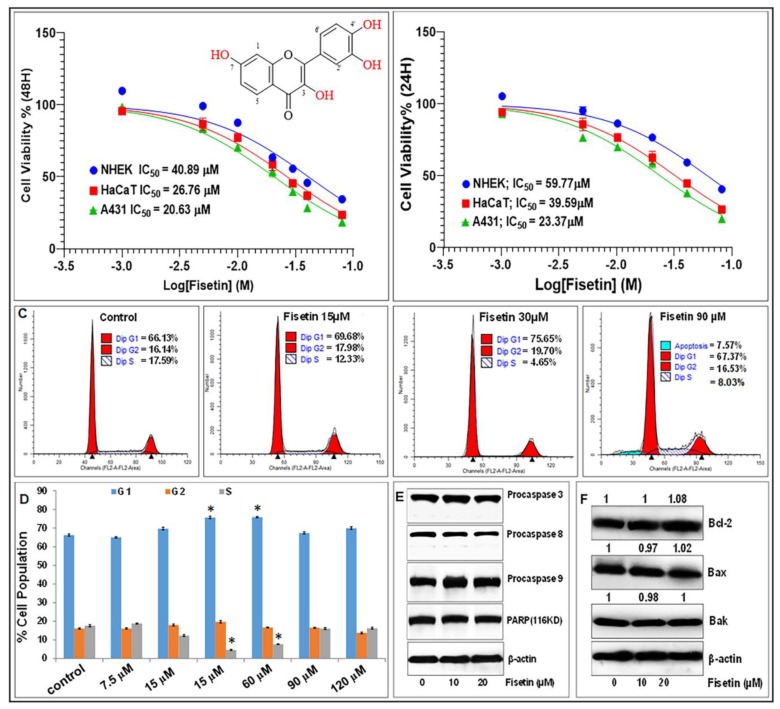
Fisetin at low doses (< 20 µM) does not significantly affect primary normal human epidermal keratinocyte (NHEK) growth and viability and does not induce apoptosis. (**A**/**B**) Relative number of viable NHEK, immortalized keratinocytes (HaCaT), and A431 cancer cells treated with or without fisetin (1–80 µM) for 24 h (**A**) and 48 h as determined by MTT assay. Mean of percentage viability of NHEK, HaCaT, and A431 cell lines plotted against the indicated doses of fisetin. Experiments were performed three times with each concentration done in octuplicate wells, and the IC_50_ values calculated from these plots are shown. (**C**) Effects of fisetin on the percentage of cells population in the different phases of cell cycle, and indication of late apoptosis or necrosis (i.e., PI-positive) cells were only seen in cells treated with higher fisetin concentration. (**D**) Levels or percentahes of cell cycle distribution in fisetin-treated cells as assessed by flow cytometry analysis. Bar graphs represent mean ± SD of results from three independent experiments performed in triplicate. Statistical significance was determined using one-way ANOVA and Dunn’s multiple comparison test and significance was considered when *p* < 0.05 (*), as compared with the control. (**E**) and (**F**) Effect of different concentrations of fisetin on the expression of markers of apoptosis including caspase-3, -8, and -9, PARP (85 kDa and 116 kDa) and Bcl-2 family of proteins (Bcl2, Bax, and Bak) on cells harvested after 48 h of treatment as analyzed by Western blotting. Equal protein loading was confirmed using β-actin as loading control. (**F**) Numerical data above the blots represent relative quantitative density values for the blots normalized with an internal loading control. The Western blot data shown are representative immunoblots of two to three independent experiments with similar results.

Dietary botanicals are important natural sources of biologically active products that possess the inherent ability to rescind multiple disease features. Fisetin (3,7,3′,4′-tetrahydroxyflavone) (Figure 1A (inset)) is a bioactive flavonol abundantly found in many dietary botanicals, particularly in pigmented fruits and vegetables, including apples, cucumbers, onions, persimmons, and strawberries [16,17,18]. Fisetin has been reported to possess pleiotropic effects in diverse disease models, including anticancer, anti-inflammatory, and antioxidant activities [19,20,21,22,23,24,25,26].

Recently, we and others, in the quest to define mechanism-based dietary antioxidants for disease prevention, showed that at higher micromolar concentrations, fisetin treatment causes growth arrest, apoptosis, and regression of both melanoma and UVB-induced cutaneous cancers by modulating the activation of components of the PI3K/Akt/mTOR signaling pathway [21,23,27]. Furthermore, we and others have recently shown that these pathways, which are frequently deregulated in diverse cancers [28,29], are also overexpressed in psoriatic and atopic dermatitis skin lesions [30,31]. There is limited knowledge regarding the role of fisetin in immune cells. In basophils, fisetin suppresses the expression level of type-2 cytokines [32]. In mice, fisetin reduces the production of type-1 and type-2 cytokines by T lymphocytes [33] and attenuates NF-κB activity and IL17 production in an in vivo allergic airway inflammation mouse model [34]. These observations led us to examine the potential of fisetin as an agent to mitigate the three major hallmarks of psoriasis: activation of inflammation, keratinocyte-induced proliferation, and aberrant differentiation [35]. To the best of our knowledge, no study has evaluated the effects of fisetin on psoriasis. In this study, we assessed the effect of fisetin in a psoriasis model, and demonstrated that at low (micromolar) concentrations fisetin inhibited intracellular PI3K/Akt/mTOR and MAPK signaling components and normal human epidermal keratinocyte (NHEK) proliferation, and promoted NHEK differentiation without inducing apoptosis. Moreover, fisetin reduced the secretion of pro-inflammatory cytokines by keratinocytes; activated peripheral blood mononuclear cells (PBMC) and CD4+ T lymphocytes; and mechanistically inhibited the intracellular PI3K/Akt/mTOR and MAPK pathways. Furthermore, the functional characteristics/roles of fisetin were also examined in an established in vivo relevant 3D full-thickness engineered human psoriasis-like skin model. Our study demonstrates that fisetin acts on both inflamed keratinocytes and immune cells in 2D and reconstituted 3D skin tissue architecture, similar to in vivo psoriatic skin lesions, and clarifies its mechanism of action in these systems.

## 2. Materials and Methods

### 2.1. Chemicals and Reagents

Fisetin, 3-[4,5-dimethylthiazol-2-yl]-2,5-diphenyl tetrazoliumbromide (MTT), propidium iodide (PI), and 12-O-tetradecanoyl-phorbol-13-acetate (TPA) were purchased from Sigma Chemical Co. (St Louis, MO, USA). The antibodies for caspases (-3, -8, and -9), PARP, Bak, Bax, Bad, Bcl-2, PathScan^®^ Multiplex (Phospho-p90RSK, Phospho-Akt, Phospho-p44/42 MAPK (Erk1/2), Phospho-S6 Ribosomal Protein, and Rab11) Western Detection Cocktail I; #5301, Phospho-p38 MAPK (Thr^180^/Tyr^182^) (D3F9) XP^®^ Rabbit mAb #4511, Phospho-Akt (Ser^473^) (D9E) XP^®^ Rabbit mAb #4060, Phospho-mTOR (Ser^2448^) (D9C2) XP^®^ Rabbit mAb #5536, Phospho-mTOR (Ser^2481^) Antibody #2974, Phospho-SAPK/JNK (Thr^183^/Tyr^185^) (81E11) Rabbit mAb #4668, β-Actin (13E5) Rabbit mAb #4970, PI3 Kinase p110α (C73F8) Rabbit mAb #4249, PI3 Kinase p85 (19H8) Rabbit mAb#4257, Phospho-Akt (Thr^308^) (D25E6) XP^®^ Rabbit mAb #13038, PhosphoPlus^®^ p70 S6 Kinase (Thr^389^, Thr^421^/Ser^424^) Antibody Kit #9430, mTOR (7C10) Rabbit mAb #2983, and Lamin B1 (D4Q4Z) Rabbit mAb #12586 were obtained from Cell Signaling Technology (Danvers, MA, USA). Recombinant human (rh) IL-22, IL-17A, TNF-α, anti-CD3, anti-CD28, and biotinylated polyclonal goat antihuman IL-17A were from R&D Systems (Minneapolis, MN, USA). Antihuman IL-17A, IFN-γ (clone 2G1) was purchased from Endogen (Pierce/Thermo Scientific, Rockford, IL, USA), IFN-γ (clone B133.5), IL-4 (clone 8D4-8) and IL-4 (clone MP-25D2) (Pharmingen, Inc., La Jolla, CA, USA), p-JNK (clone G-7,sc-6254), p-p38 (clone D-8, sc-7973), filaggrin (clone AKH1,sc-66192), p-p38(sc-7973), cytokeratin-1(sc-65999), cytokeratin-10 (sc-51581), Transglutaminase 1 (sc-25786), Fra-1(sc-605X), c-Fos(sc-52X), Fos B(sc-8013), c-Jun(sc-1694), Jun B(sc-46x), Jun D(sc-74), caspase-14 (sc-5628), were all obtained from Santa Cruz Biotechnology, Inc. (Santa Cruz, CA, USA). CELLnTEC progenitor cell culture medium was from ZenBio (ZenBio, Raleigh, NC, USA). Fetal bovine serum (FBS) was obtained from Life Technologies (Grand Island, NY, USA). The transglutaminase activity assay kit (C, K571-100) was from BioVision, Inc. (Milpitas, CA, USA). The Procarta Plex Mix & Match Human 6-Plex kit was from eBioScience/Affymatrix (EPX060-15073; Santa Clara, CA, USA). Horseradish peroxidase conjugated antimouse or antirabbit secondary antibody was obtained from Amersham Life Science, Inc. (Arlington Heights, IL, USA), and the BCA protein assay kit was obtained from Pierce (Rockford, IL, USA). Novex precast Tris-glycine gels were obtained from Invitrogen (Carlsbad, CA, USA).

### 2.2. Human Subjects

The study protocols were approved by the University of Wisconsin-Madison Health Sciences Institutional Review Board protocols #2013-0059-CR004 and 2013-1570, and informed written consent was obtained from subjects prior to participation. The study was conducted according to the principles of the Declaration of Helsinki.

### 2.3. Keratinocyte Isolation, Culture, Activation with IL-22, TNF-α, TPA, and Treatments

Primary normal human epidermal keratinocytes were isolated from neonatal foreskin and adult skin biopsies, and primary cultures were established in CELLnTEC progenitor cell culture medium (ZenBio, Raleigh, NC, USA) supplemented with penicillin (100 U/mL), 100 μg/mL streptomycin (100 μg/mL), and amphotericin (100 μg/mL) (Life Technologies), as previously described [36,37]. Frozen cells were thawed and maintained for about two months (~8 passages). Human epidermoid carcinoma A431 cells, obtained from ATCC (Manassas, VA, USA), and human immortalized keratinocytes HaCaT cells from Life Technologies Corporation (ThermoFisher Scientific, Waltham, MA, USA) were cultured and maintained in DMEM, supplemented with 10% FBS and 1% penicillin–streptomycin. Stock solutions of fisetin were made in dimethyl sulfoxide (DMSO), and further diluted in the respective growth media for the treatment of NHEK, A431, and HaCaT cells. Control cells were treated with an equivalent volume of the vehicle alone, corresponding to a final maximum concentration of 0.1% (v/v) DMSO for each treatment, at which point the concentration had no effect on the cell viability. All cells were maintained at 37 °C in a humidified atmosphere of 95% air and 5% CO_2_, and the growth media were replenished every alternate day until reaching desired confluence (60–80%) prior to experimentation. For rhIL-22 stimulation and drug treatment protocol, near-confluent keratinocytes were pretreated with or in the absence of varied concentrations of fisetin (10–20 μM) for 8 h, followed by co-treatment with or without rhIL-22 (20 ng/mL) for 40 h (making a total of 48 h exposure to fisetin). Cells were harvested and lysates were prepared for immunoblot as described below. IL-22-induced cell proliferation and viability analysis in the presence or absence of fisetin were as described below.

For TNF-α treatment, cells were cultured and pretreated with fisetin overnight and were stimulated with or without rhTNF-α (10 ng/mL) for 10 to 60 min prior to harvesting for Western blotting analysis, as described below. For 12-*O*-tetradecanoyl-phorbol-13-acetate (TPA) stimulation, near-confluent keratinocytes were pretreated with or without altered concentrations of fisetin (10–20 μM) for 20 h, followed by co-treatment with or without 100 nM/mL of TPA for the final 6 h (for a total of 26 h of exposure to fisetin). Cultured supernatants were centrifuged and centrifugates were collected and stored at −80 °C until used. The supernatants were only subjected to a single freeze‒thaw cycle and used for the analysis of TPA-induced keratinocytes secreted pro-inflammatory mediators (IL-1α, IL-1β, IL-6, IL-8 (CXCL8), TGF-α and TNF-α according to the manufacturer’s protocol and as previously described [38], and as described below.

### 2.4. Determination of Cell Viability by MTT Assay

The effect of fisetin on the viability of cells was determined by 3-[4,5-dimethylthiazol-2-yl]-2,5-diphenyl tetrazoliumbromide (MTT) assay. NHEK, A431 and HaCaT cells were plated at 5 × 10^3^ cells per well in 200 μL of respective culture media in 96-well microtiter plates. After the cells were adherent and proliferating, the media containing fisetin (0–80 μM) were replenished and cultured for 24 or 48 h. After incubating for the specified times, MTT solution (0.5 mg/mL in phosphate-buffered saline (PBS)) was added to each well and incubated for 3 h, after which the plate was centrifuged at 2000 rpm for 5 min at 4 °C.

Additionally, the effect of fisetin treatment on the viability of NHEK was assessed in relation to the proliferative effect following prestimulation with or without rhIL-22 (20 ng/mL) and was determined by MTT assay. Briefly, NHEKs were seeded at a density of 2 × 10^4^ cells/well in 24-well poly(D)lysine (0.1 mg/mL; Sigma-Aldrich, St. Louis, MO, USA) precoated plates in 1 mL complete culture medium and incubated at 37 °C and 5% CO_2_. At 80% confluence, cells were treated for 6 h with/without fisetin 10–20 μM, after which rhIL-22 (20 ng/mL) was added and further cultured. At 48 h of incubation, the medium was removed and cells were rinsed with PBS and incubated for 3 h with 150 μL of MTT solution (0.5 mg/mL in medium). In either case, the MTT solution was removed and the formazan crystals were then solubilized in DMSO (200 μL) by shaking, and absorbance was spectrophotometrically recorded at 570 nm on a Bio-Tek microplate reader (Bio-TEK Instruments Inc., Winooski, VT, USA). The experiment was repeated three times each sample performed in sextuplicate with similar results. The effect of fisetin on normal and IL-22 stimulated growth inhibition was assessed as percentage cell viability, where DMSO-treated cells (untreated controls) were considered 100% viable. DMSO at the concentrations used has no effect on cell viability.

### 2.5. Cell Cycle Analysis

NHEKs were treated with fisetin (1‒120 μM) for 24 h, harvested, and fixed in chilled 70% alcohol overnight. Cells were then washed twice with PBS, digested with DNase-free RNase (10 μg/mL) at 37 °C for 1 h, stained with PI (5 μg/mL) for 3 h at 4 °C in the dark, and analyzed by FACS Calibur (Becton Dickinson, Franklin Lakes, NJ, USA) for cell cycle phase distribution.

### 2.6. Preparation, Culture, Activation, and Treatment of Peripheral Blood Mononuclear Cells (PBMC)

Peripheral blood was obtained by venipuncture from volunteer healthy donors with appropriate University of Wisconsin-Madison Health Sciences Institutional Review Board approval, as previously described, with some modifications [39]. Briefly, heparinized whole blood was centrifuged (700× *g*, 20 min) over a Percoll density gradient (density 1.090 g/mL; Pharmacia Biotech, Piscataway, NJ, USA) to separate mononuclear cells from granulocytes. PBMCs were then washed, layered over new calf serum, and centrifuged at 800 rpm for 15 min to eliminate platelets. PBMCs were cultured in 24-well dishes at 1.0 × 10^6^ cells/mL with RPMI 1640, 10% FBS, and antibiotics. PBMCs were treated with fisetin (10 μM) for 10 min, and then activated with anti-CD3 (R&D Systems; coated on plates at 1 μg/mL) plus soluble anti-CD28 (R&D systems; 1 μg/mL). After 6 h of culture, RLT buffer (Qiagen, Valencia, CA, USA) was added on cells for RNA extraction and PCR analysis. After 48 h of culture, cells’ supernatant fluids were harvested for the measurement of proteins by ELISA.

### 2.7. Preparation and Activation of Blood CD4+ T Lymphocytes

The CD4+ T lymphocytes were prepared as previously described [40]. Briefly, heparinized blood was diluted 1:1 in HBSS, and overlaid on Percoll (1.090 g/mL). After centrifugation at 700× *g* for 20 min at room temperature, the mononuclear cells were recovered from the plasma/Percoll interface, and CD4+ cells were prepared by negative selection using the Miltenyi Biotec (San Diego, CA, USA) CD4^+^ T Cell Isolation Kit II. Using this method, purity was typically >98% as determined by flow cytometry [40]. For activation, CD4^+^ T cells (2 × 10^6^/mL) were cultured in 1 mL of complete medium (RPMI plus 10% FBS) with 1 μg/mL of plate-bound anti-CD3 plus 1 μg/mL of soluble anti-CD28 (clones 37407 and UCHT1, respectively; R&D Systems, Minneapolis, MN, USA) in a 24-well plate (Corning Costar, Lowell, MA, USA). In this condition, after 48 h activation, CD4^+^ T cells had a memory/effector phenotype (CD45RO+ CD25+) as previously shown [40] and were placed with the tissue-engineered reconstructions over it. CD4+ T cell subset purification was performed by positive selection with magnetic bead separation (Miltenyi), and cell and FTRHSP-free supernatants’ cytokine levels were detected by ELISA, as above.

### 2.8. ProcartaPlex^TM^ Multiplex Bead-Based Immunoassays for Cytokines and Chemokines

Cultured conditioned supernatant from TPA-stimulated and control keratinocyte cultures in the presence or absence of varied concentration of fisetin were used to determine the levels of produced cytokine and chemokine levels by human 6-Plex Procarta Multiplex Beads. A commercially available human Procarta 6-Plex Bead immunoassay kit (Affymetrix/eBioscience) was used to determine cytokine and chemokine levels in conditioned media from TPA-stimulated NHEKs as described earlier [38]. The assay includes a range of six customized human pro-inflammatory cytokine and chemokine panels that reflect key processes and responses relating to the activation of keratinocyte inflammation. Beads of defined spectral properties conjugated to analyte-specific capture antibodies were combined with 50 μL of cultured supernatant samples to be tested in separate wells of a 96-well black side/transparent bottom microplate and incubated at room temperature (RT) for 120 min following the manufacturer’s instructions and as previously described [38]. All cell culture supernatant samples were analyzed in triplicate along with serial standards (7-point dilutions). After each analyte was allowed to bind to the captured antibodies on the beads, washes were performed to remove nonspecifically bound proteins, and analyte-specific biotinylated detection antibodies were added and incubated with the beads at RT for 30–60 min. During this incubation, the analyte-specific biotinylated detection antibodies bind to specific epitopes on the immobilized analytes. Following the binding incubation and washes, streptavidin conjugated to phycoerythrin (SA-PE or streptavidin-PE), a pigment complex that serves as a fluorescent tag, was added, and samples were incubated at RT with slow shaking for 30 min. During this final incubation, streptavidin-RPE binds to the biotinylated detector antibodies associated with the immune complexes on the beads, forming a four-member solid phase sandwich. After washing cycles to remove unbound SA-PE, excess reading buffer was added and incubated at RT for a minimum of 5 min in the dark with shaking and stored at 4 °C overnight. Fluorescently tagged beads were analyzed with the xMAP Luminex reader (Luminex, Austin, TX, USA) for quantitative analysis. The spectral properties of the beads and the amounts of associated PE fluorescence were monitored to determine the concentration of the analytes IL-1α, IL-1β, IL-6, CXCL8 (IL-8), TNFα, and TGF-α in cultured supernatants from different NHEK treatment groups. The analyte concentrations were determined by importing the data into Procarta Plex^TM^ Multiplex Analyst software v.1.0 (Affymetrix/eBioscience) for analysis, as described earlier [38]. The cytokine/chemokine values obtained by immunoassay were referenced against established standards to allow for comparisons between the study groups.

### 2.9. ELISA Analysis of Th1-Th2-and-Th17 Cytokines, IL-4, IL-17A, and IFN-γ

Supernatant cultures from antiCD3/antiCD28-stimulated PBMC in the presence or absence of varied concentrations of fisetin were used to determine the levels of produced pro-inflammatory cytokine levels by a sandwich ELISA immunoassay. Cytokines were measured in the 48 h PBMC and CD4+ T cells’ supernatant fluids. IL-17 A, IFN-γ, and IL-4 levels were measured by utilizing an “in-house” sandwich ELISA [40,41]. Coating antibodies included antihuman IL-17A (clone 41809.111, R&D Systems), IFN-γ (clone 2G1, Endogen/Pierce/Thermo Scientific), and IL-4 (clone 8D4-8, Pharmingen, Inc.). Detection antibodies included biotinylated polyclonal goat antihuman IL-17A (R&D Systems), IFN-γ (clone B133.5, Pharmingen, Inc), and IL-4 (clone MP-25D2, Pharmingen, Inc). The ELISA assay sensitivities were < 5 pg/mL for others and < 3 pg/mL for IL-17A.

### 2.10. RNA Preparation and Real-Time Quantitative (q)-PCR

Total RNA was extracted from PBMC using the RNeasy Mini Kit (Qiagen). The reverse transcription reaction was performed using the Superscript III system (Invitrogen/Life Technologies, Grand Island, NY, USA). Gene expression levels were determined by qPCR using SYBR Green Master Mix (SA Biosciences, Frederick, MD, USA). Forward and reverse specific primers ((IFNG), forward: gaaacgagatgacttcgaaaagct, reverse: catgtattgctttgcgttgga, and (IL17A), forward: cgatccacctcaccttgga, reverse: tcccagatcacagagggatatctctc) were designed using Primer Express 3.0 (Applied Biosystems, Carlsbad, CA, USA) to span an exon‒exon junction, and blasted against the human genome to determine the specificity using the National Center for Biotechnology Information - NCBI primer blast (http://www.ncbi.nlm.nih.gov/tools/primer-blast). The reference gene primers, β-glucuronidase ((GUSB), forward: caggacctgcgcacaagag, reverse: agcgtgtcgacccattc), were used to normalize the samples. Standard curves and efficiencies were determined for each set of primers. Efficiencies ranged between 91% and 96%. Data are expressed as a fold change using the comparative cycle threshold (∆∆CT) method, as described previously [40]. The values presented are fold change = (2^−∆∆Ct^) compared to expression in resting PBMC.

### 2.11. Preparation of Protein Lysates from Cultured Cells and Western Blotting

Following the treatment of NHEK cells with fisetin (10–20 μM; for a maximum of 48 h prior to harvest), in the presence or absence of TNF-α or rhIL-22, the media was aspirated, and the cells were washed with cold PBS (pH 7.4). Cells were incubated in ice-cold lysis buffer (50 mM Tris-HCl, 150 mM NaCl, 1 mM ethyleneglycol-bis(aminoethylether)-tetraacetic acid (EGTA), 1 mM EDTA, 20 mM NaF, 100 mM Na3VO4, 0.5% NP-40, 1% Triton X-100, 1 mM phenylmethylsulfonyl fluoride (PMSF) (pH 7.4), with freshly added protease inhibitor cocktail (Protease Inhibitor Cocktail Set III, Calbiochem, La Jolla, CA, USA) on ice for 30 min. The cells were scraped and cell lysates collected in microfuge tubes and passed through 22.5-gauge syringe needles to break up the cell aggregates. The lysates were cleared by centrifugation at 14,000 *g* for 30 min at 4 °C, and the supernatant (whole cell lysate) protein concentrations were determined using a BCA protein assay kit (Pierce) according to the manufacturer’s protocol. Lysates were used or immediately aliquoted and stored at −80 °C for further analysis. Western blotting was performed as previously described [30,42]. Briefly, 10‒20 μg protein was resolved on 4–12% polyacrylamide gels and transferred to a nitrocellulose membrane. The blots were blocked in blocking buffer (5% nonfat dry milk or BSA/1% Tween 20; in 20 mM TBS, pH 7.6) for 45 min at room temperature. Membranes were incubated with an appropriate monoclonal or polyclonal primary antibody in the blocking buffer for 2 h at RT, to overnight at 4 °C, followed by 3 × 5-min washes, and incubated with antimouse or antirabbit horseradish peroxidase-conjugated (HRP) secondary antibody obtained from Amersham Life Science Inc. Chemiluminescence and autoradiography was detected using Bio-Rad detection and analysis systems, as described earlier [30,42]. Equal loading of protein was confirmed by stripping the immunoblot and re-probing for β-actin, vinculin or lamin B1 as loading controls. The immunoblot results displayed herein are representative of three independent experiments.

### 2.12. Generation of a Three-Dimensional (3D) Full-Thickness Reconstituted Human Skin Model of Psoriasis (FTRHSP)

A metabolically active 3D full-thickness reconstructed human skin model of psoriasis (FTRHSP) was generated as described previously [36,37], with slight modifications. The FTRHSP consisted of a multilayered NHEK in a highly differentiated epidermis developed on a fibroblast- contracted collagen dermal substratum, and a suspension of activated CD4+ T cells underneath the 3D reconstructed epithelium exposed at the air‒liquid interface (ALI) for 12‒14 days as described previously [36,37], with slight modifications. The dermal component was prepared by contracting collagen gels populated with fibroblasts seeded into 3.0 μm Millicell-PCF inserts (Millipore Corporation, Billerica, MA, USA), each placed in wells of a 24-well plate as described previously, and the epidermal component was generated on top [37,43]. For epidermal components, second- to third-passage NHEKs established in low-calcium Epi-Life medium were seeded, synchronized, and maintained in progenitor cell targeted culture media (CnT-02-07; CELLnTEC, ZenBio, Research Triangle Park, NC, USA) prior to harvesting and used to generate the 3D FTRHSP cultures as described earlier [37,43]. Briefly, after trypsinization, 3 × 10^5^ cells per insert in 250 μL of CnT-02-07 were seeded into the surface of the generated dermal substratum, each placed in wells of six-well tissue culture plates containing 2.5 mL of CnT-02-07 medium in a humidified atmosphere with 5% CO_2_ and maintained at 37 °C. After 48 h of culture, CnT-02-07 was switched to CnT-02-3DP5 differentiation medium (2 mL outside the insert, and 0.4 mL inside the inserts (day 2)), and further grown for 48–72 h to initiate differentiation (day 5). The media outside and inside the inserts were then removed, and the medium outside the insert was replenished with 1.2 mL of CnT-02-3DP5 complete medium and cultured at ALI from this time point onwards. After fivedays of cultures at ALI, the medium outside was replenished with prewarmed CnT-02-3DP5 medium in plates containing coated anti-CD3 and soluble anti-CD28 stimulated CD4+ T cells (3 × 10^5^/0.9 mL) (herein referred to as activated T cells), prepared as above (day 10) to initiate inflammation. Anti-CD3/CD28 CD4+ T cells in 0.9 mL of medium in the transwells at day 10 were overlaid by placing the insert on top, exposing the underneath dermal side of the 3D skin equivalents on it. Memory T cells were activated, while control or inactivated CD4+ T cells were employed in the control reconstructs. On the same day of co-culture of 3D and activated T cells, topical treatment with or without different concentrations of the test compounds (fisetin or Vit-D_3_) was initiated. Fresh media were replenished as above every other day and continued prior to harvest at the indicated harvest day (day 15). After five days of co-culture in the presence or absence of the test compounds, and at harvesting, 3‒4 mm diameter punch biopsies from each insert were obtained, formalin-fixed, and processed for either H&E or immunostaining. We then analyzed the morphology and protein expression levels of markers of inflammation, proliferation, and differentiation as well as activation of the PI3K/Akt/mTOR cascade. Additionally, cell-free supernatants were collected for pro-inflammatory cytokine analysis using sandwich ELISA as described above.

### 2.13. Fisetin and Vit-D3 Treatment of the 3D FTRHSP Model System

Fisetin and Vit-D_3_ were dissolved in DMSO and stored as 80 mM, and 0.1 mM aliquots, respectively, at −20 °C protected from light. At the time of use, test compounds, fisetin (1–80 μM), or vit-D_3_ (0.1 μM) were diluted in a culture medium containing 0.1% BSA to stabilize the drugs, and treatment was started at 80% confluence (for monolayer). For the 3D FTRHSP studies exposed to ALI, 10 μL of different concentrations of the test agents, fisetin (10–40 μM) and vit-D_3_ (1 × 10^−7^ μM), which are known to control psoriasis, were added to the developing tissue.

### 2.14. Histology, Morphometry, and Immunostaining Analyses of the FTRHSP

Formalin-fixed, paraffin-embedded (FFPE) 5-μM cross sections were de-paraffinized by incubation in xylene (soaking twice for 10 min each), rehydrated with ethanol (twice in 100% ethanol for 10 min, twice in 95% ethanol for 10 min and then in 70% ethanol for 10 min), and finally placed in distilled water for 10 min. Antigen retrieval was performed by treatment with fresh sodium citrate buffer with 0.05% Tween 20 at pH 6.0, for 30 min at 100 °C, and sections were stained with H&E and evaluated for gross morphology, epidermal, and horny layer thickness and immunostained for the specified markers as previously described [37,44,45]. Briefly, endogenous peroxidase was blocked with 3% H_2_O_2_ in methanol and incubated with a blocking solution containing 3% bovine serum albumin ( BSA)/3% normal goat serum (NGS)/0.4% Triton X-100 in PBS for 60 min at RT, followed by overnight incubation at 4°C with monoclonal antibodies (mouse cytokeratin 10 (RKSE 60) (1:75, sc-23877), mouse anti-Desmoglein-1 (1:75, Invitrogen #326000), rabbit anti-filaggrin (1:100; ab34584); rabbit anti-involucrin (1:75; 2215)). After three washes, samples were incubated for 2 h with Texas Red@-X Goat Anti-mouse (1:600; Invitrogen T6390) and Alexa fluor Goat Anti-rabbit, (1:600; Life Technology A11008), followed by three washes of 10 min each, and were mounted in situ mounting media with 4′,6-diamidino-2-phenylindole (DAPI) (#DUO82040, Sigma). Slides were visualized on an Olympus IX71 system microscope (Olympus/HuntOptic & Imaging, Inc., Pittsburgh, PA, USA), and digital images at 20× magnification were captured with a Olympus U-CMAD3 adaptor attached Olympus DP71 camera (Olympus/HuntOptic & Imaging, Inc.) linked to a high-resolution computer screen and connected to an Olympus U-RFL-T Mercury Burner. Images were processed using the Olympus CellSens dimension imaging software v1.6. The thickness in terms of the area of the entire epidermis and the stratum corneum were determined as described earlier [37].

### 2.15. Statistical Analysis

Statistical analyses were carried out with GraphPad Prism version 7.1 (San Diego, CA, USA), except for qPCR and ELISA, which were performed using the SigmaPlot 11.0 software package (Systat Software, Inc., San Jose, CA, USA). All quantitative data are expressed as means ± SD or SEM, and significant differences were determined by the Student’s *t*-test or ANOVA with Bonferroni and/or Tukey post hoc testing; *p* values < 0.05 were considered significant.

## 3. Results

### 3.1. Fisetin Inhibits Cell Proliferation and Viability, but Does Not Affect Apoptosis of Keratinocytes at Doses ≤20 µM

We first examined the effect of fisetin on the growth and viability of primary NHEK compared to HaCaT, a precancerous immortalized human keratinocytes and A431, an epidermoid carcinoma cell line. Employing the 3-(4,5-dimethylthiazol-2-yl)-2,5-diphenyltetrazolium bromide (MTT) assay, dose-and-time course analyses revealed that after 24 and 48 h, fisetin at doses ≤20 µM had modest effect on NHEK, while exerting significant inhibition of cell growth and viability of HaCaT and A431. The IC_50_ on NHEK were calculated to be 59.8 µM and 40.9 µM, as compared to HaCaT of 39.6 µM and 26.8 µM and A431 of 23.4 µM and 20.6 µM at 24 h and 48 h, respectively (Figure 1A,B).

To examine whether the effect of fisetin on viable cell number is related to cell cycle arrest or apoptosis, the proportion of cells containing diploid (sub-G_1_) levels of DNA was quantified using propidium iodide (PI) staining and flow cytometric analysis. Treatment with 90 and 120 µM of fisetin as a positive control for apoptosis [46] significantly increased the proportion of NHEK in the sub-G_1_ population (6.9% and 8.24%) compared to lower doses of fisetin (30–60 µM) that only expanded the G_1_ and increased the S-phase compartments (0% and 0.22%), but did not induce any increases in keratinocyte apoptosis (Figure 1C,D). Fisetin at 5–30 µM did not exhibit toxicity, but cell death was evident at higher doses (Figure 1C,D). Western blotting revealed that, at concentrations less than or equal to 20 µM, fisetin did not induce apoptosis at the time points investigated, as evidenced by the absence of activation of caspases 3, 8, and 9 and/or changes in PARP, Bak, Bax, and Bcl2 protein levels (Figure 1E,F). These observations led us to select doses of 0 to 20 μM over 24–48 h for further mechanistic studies.

### 3.2. Fisetin Treatment Promotes Human Primary Epidermal Keratinocyte Differentiation and Upregulates the Expression of AP-1 Transcription Factor Proteins

**Figure 2:**
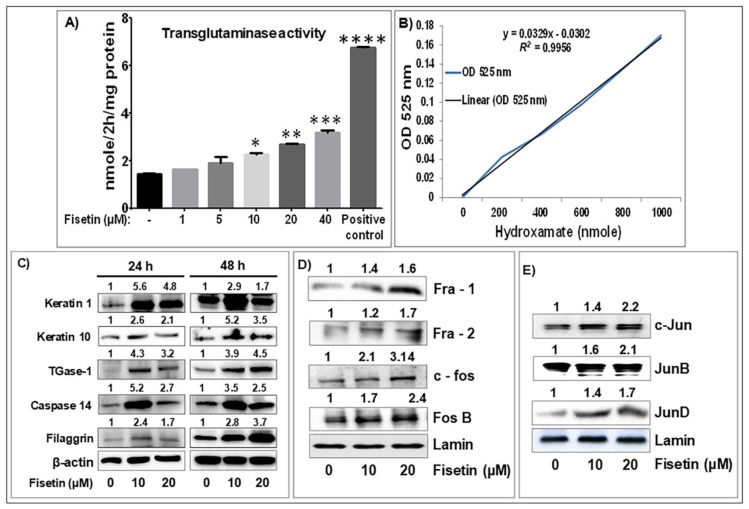
Fisetin treatment increased the expression of markers of epidermal keratinocytes’ differentiation and AP-1 transcription factor in NHEK. (**A**,**B**) The bar graphs represent dose-dependent induction of the differentiation marker transglutaminase (TGase) (a terminal differentiation marker) activity in NHEK treated with or without different doses of fisetin for 24 h, and the linear plot of the hydroxamate analysis of TGase activity. Data represent means ± SEM of three independent experiments, each performed in quadruplicate. Significance was assessed for control cells versus fisetin-treated cells or positive control, determined by one-way analysis of variance, as denoted by * *p* < 0.05, ** *p* < 0.01, *** *p* < 0.001, and **** *p* < 0.0001. Dose- and time-dependent increases in the protein expression of (**C**) early and late differentiation markers, and (**D**,**E**) nuclear protein expression of members of AP-1 factors subunits including Jun (C-Jun, Jun B, Jun D) and Fos (C-Fos, Fra-2, Fos-B) in NHEKs when compared to control cultures after 48 h, as analyzed by Western blotting. Panels (**C**–**E**) are representative of 2–3 experiments with similar results. Equal protein loading was confirmed using β-actin and lamin as loading controls, and the numerical data above the blots represent the quantitative relative densitometry values for the blots normalized to their respective loading controls.

Since psoriasis is characterized by aberrant keratinocyte differentiation, a key requirement for epidermal homeostasis [10], we examined whether the antiproliferative effect of fisetin on NHEK paralleled differentiation. The ability of different concentrations of fisetin to induce terminal differentiation was analyzed by monitoring transglutaminase (TGase) enzyme activity, a marker of terminal keratinocyte differentiation. Significant dose-dependent induction of TGase activity was observed in NHEK (Figure 2A,B (graph for linear regression used to plot TGase activity)). In addition, the ability of fisetin to stimulate differentiation was examined by Western blot analysis of the expression levels of early (K1 and K10), intermediate (caspase-14) and late (filaggrin and TGase-1) keratinocyte differentiation markers [36,47,48]. Following 24–48 h exposure of near-confluent NHEK, fisetin dose- and time-dependently increased the expression of these differentiation markers (Figure 2C), indicating that fisetin induces terminal differentiation of keratinocytes at higher concentrations.

Activator protein-1 (AP-1), a dimeric protein complex comprised of members of the Jun and Fos proto-oncogene subfamilies, is a transcription factor known to play an important role in the regulation of epidermal keratinocytes proliferation, terminal differentiation, cytokine production, and inflammation [49,50,51]. AP-1 subunits’ expression levels are downregulated in lesioned compared with nonlesioned psoriatic skin [52,53]. We determined the effect of fisetin on the expression of AP-1 factors in NHEKs and observed that fisetin increased the nuclear expression of members of AP-1 factor subunits, including Fos (Fra-1/2, c-Fos, Fos B) and Jun (c-Jun, JunB, JunD), as compared to control cultures (Figure 2D,E).

### 3.3. Fisetin Regulates IL-22-Induced Keratinocyte Proliferation by Inhibiting the PI3K/AKT and mTOR Pathway Components

It is known that type-17 cytokines such as IL-17 and IL-22 play critical roles in psoriasiform pathology [54,55], including Akt/mTOR activation [56,57]. In this context, the modulation of PI3K/Akt/mTOR activity is known to promote epidermal hyperplasia and exert distinct regulatory roles in the same innate immune cells that are implicated in the immunopathogenesis of psoriasis [31,58,59]. Here, we employed recombinant human (rh) IL-22-activated keratinocytes in vitro to investigate the mechanistic role of fisetin in psoriasis-like changes driven by epidermal keratinocytes hyperproliferation. We observed by MTT analysis that IL-22 treatment of NHEK significantly induced proliferation (117 ± 0.16%) compared to the control (100 ± 0.11%) (Figure S1, *p* < 0.01). When NHEK cultures were treated with 10 and 20 µM fisetin for 24  h before stimulation with 20 ng/mL of rhIL-22, IL-22-induced proliferation (117 ± 0.16%) was significantly reduced to 75 ± 0.54%, and 65 ± 1.09%, respectively (Figure S1, *p* < 0.01).

**Figure 3:**
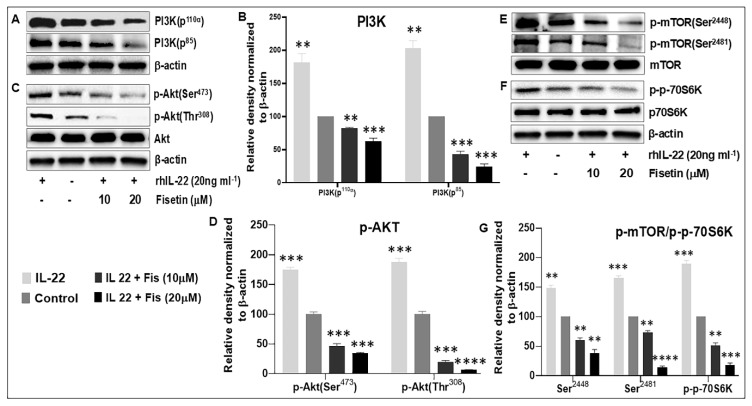
Fisetin significantly suppresses the IL-22-induced activation of PI3K/Akt/mTOR signaling in NHEK. Western blot determined protein expression levels of (**A**,**B**) PI3-K (p110 and p85), and (**C**,**D**) phosphorylation of Akt (at Ser473 and Thr308), and (**E**) phosphorylation of mTOR (at Ser2448 and Ser2481), and (**F**) phosphorylation of p-p70S6K (Thr389), as compared with untreated cells (control) and IL-22-treated cells only. (**B**,**D**,**G**) graphs of the relative intensity of normalized protein components, where each bar depict means +/-SD of three different experiments. **, *** and **** indicate *p* < 0.01, *p* < 0.001, and *p* < 0.0001 vs. control for IL-22-treated only or vs. IL-22 for fisetin-treated cells.

Studies have demonstrated that IL-22-induced proliferation of NHEK is mediated through the PI3K/AKT/mTOR signaling pathway [38,56,60]. Therefore, to mechanistically elucidate how fisetin inhibits the proliferative responses, we examined the effects of fisetin on the IL-22-induced activation of PI3K, Akt, mTOR, and p70S6K after 12 h. Our Western blot analysis showed that fisetin treatment of NHEK significantly and dose-dependently suppressed IL-22-induced increases in the protein expression of PI3Ks (p110α and p85) and the phosphorylation of Akt (at Ser^473^ and Thr^308^) and of mTOR (at Ser^2448^ and Ser^2481^), as well as its downstream effector p-p70S6K (Thr^389^), as compared with the control (Figure 3).

### 3.4. Fisetin Regulates TNF-α-Induced Activation of the PI3K/Akt/mTOR and MAPK Signaling Pathways in NHEK

**Figure 4:**
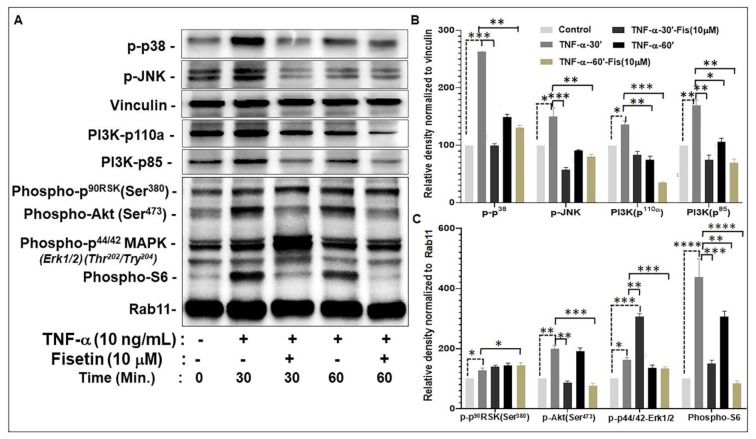
Fisetin regulates the PI3K/Akt/mTOR and MAPK signaling pathways in TNF-α-activated NHEK. NHEK cultures were pretreated for 24  h with/without 10 μM fisetin and then stimulated with 10 ng/mL TNF-α for 30 or 60 min prior to immunoblot analysis and quantification. (**A**) Western blot bands showing the protein expression levels of; p-p38, p-JNK, PI3K (p110a and p85), p-p90RSK, p-Akt(Ser473), p-ERK(p44/42) and (phospho-S6). Proteins were quantitated using the Bio-Rad Image Lab v6 software compared with untreated and TNF-α-activated control cells and normalized to Rab11 or vinculin as loading controls. (**B**,**C**) Quantitative analysis of normalized target protein expressions, and the plotted values are mean ± SD of each dataset from three independent experiments performed in triplicates. Significance of comparisons were made for TNF-α-stimulated control cells versus fisetin-treated at the 30 and 60 min time points, corresponding to solid lines, and also between unstimulated control cells versus TNF-α-stimulated cells indicated by the broken lines, as denoted by * p < 0.05, ** p < 0.01, *** p < 0.001, and **** p < 0.0001.

Given that fisetin inhibited IL-22-induced responses that are dependent on PI3K/AKT/mTOR and MAPK signaling, we next examined the effect of fisetin on TNF-α-induced PI3K/AKT/mTOR and MAPK pathway responses. NHEK cultures were pretreated with 10 µM fisetin for 24 h before stimulating with 10 ng/mL of rhTNF-α, and the ensuing effect on TNF-α-induced activation of PI3K/AKT/mTOR and MAPK- signaling was examined by Western blotting after 30 and 60 min (Figure 4). At 30 min, TNF-α treatment increased the levels of p-p38, p-JNK, PI3K (p110a and p85), p-p90RSK, p-Akt, p-ERK, and p-S6 (Figure 4). These TNF-α-induced effects were significantly inhibited upon pretreatment with 10 μM fisetin, except for the expression of p-p90RSK and p-ERK, the latter of which was enhanced by fisetin treatment (Figure 4).

### 3.5. Fisetin Pretreatment Inhibits Human Epidermal Keratinocytes, Peripheral Blood Mononuclear Cells (PBMC), and CD4+ T-Lymphocytes-Induced Inflammatory Responses

**Figure 5:**
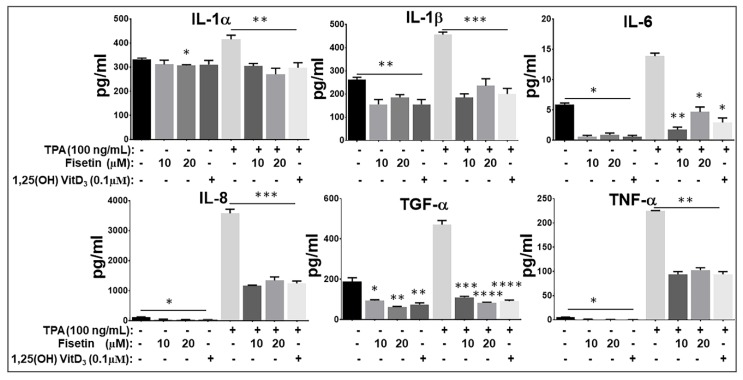
Fisetin reduces the inflammatory response in 12-O-tetradecanoyl-phorbol-13- acetate (TPA)-activated NHEK. NHEKs were pretreated with or without different concentrations of fisetin (10 and 20 µM) for 24 h, before stimulation with or without 100 ng/mL of TPA. The release of pro-inflammatory cytokines (IL-1 α, IL-1 β, IL-6, IL-8 (CXCL8), TNF-α, and TGF-α) in condition culture media was measured by Procarta-based multiplex immunoassay. Fisetin pretreatment suppressed TPA-induced secretion of these proinflammatory mediators to values similar to that of 1, 25 dihydroxyvitamin D3 (Vitamin D3) pretreatment. Values are the mean ± SD of results from three independent experiments each performed in quadruplicate. Statistical significance was determined using one-way ANOVA and Tukey’s multiple comparison test. Significance of comparisons were made for TPA-stimulated alone cells versus TPA-stimulated and fisetin/vitamin D_3_-treated cells, and also between unstimulated control cells versus fisetin/vitamin D3-treated cells, as denoted by * p < 0.05, ** p < 0.01, *** p < 0.001, and **** p < 0.0001.

Next, we examined the role of fisetin on inflammatory responses induced by 12-*O*-tetradecanoylphorbol 13-acetate (TPA) on NHEK and induced by anti-CD3 plus anti-CD28 on PBMCs, and CD4+ T lymphocytes. Using Procarta-based Multiplex cytokine immunoassay, we first examined the effects of fisetin (10 and 20 μM) pretreatment on the TPA (100 ng/mL)-induced production of pro-inflammatory mediators IL-1α, IL-1β, IL-6, IL-8, TGF-α, and TNF-α by NHEK (Figure 5). It has been shown that treatment of NHEK with TPA can induce an inflammatory response mimicking the initial keratinocyte activation phase of the psoriatic process [10,61]. Here, we observed that fisetin pretreatment significantly decreased inactivated and TPA-induced NHEK secretion of the proinflammatory cytokines IL-1α, IL-1β, IL-6, IL-8, TNF-α, and the profibrotic mediator TGF-α. Of note, fisetin’s effects were highly similar to the effects elicited by 1, 25 hydroxyvitamin-D_3_ (Vit-D_3_) treatment upon TPA stimulation (Figure 5).

**Figure 6:**
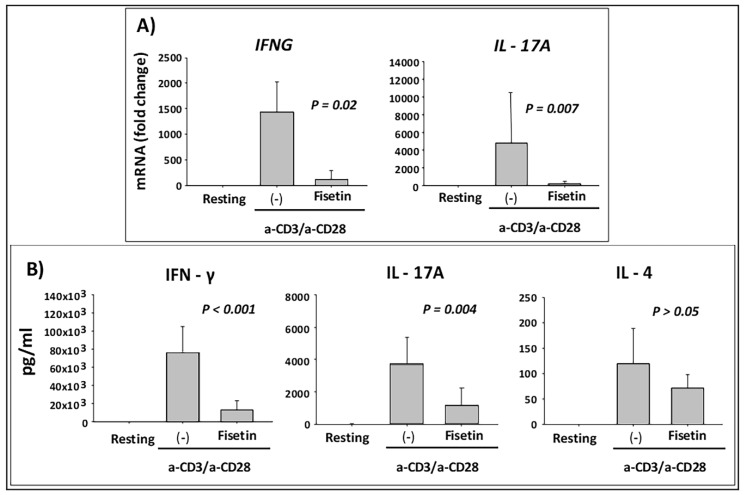
Fisetin suppresses the expression and secretion of IFN-γ and IL-17 in activated human peripheral blood mononuclear cells (PBMC). PBMC were prepared from the circulating blood from three different healthy blood donors, and were cultured without (resting) or with anti-CD3 plus anti-CD8 (a-CD3/a-CD28), either treated with 10 µM of fisetin or the vehicle alone (-) for 10 min before activation. (**A**) PBMC were activated for 6 h before measurement of the type-1 and type-17 proinflammatory cytokines IFN-γ (*IFNG*) and IL-17A (*IL17A*) mRNA expression levels. Fold change in mRNA levels compared to resting cells, whose expression level was fixed at 1, were determined. Means ± SE are shown, and log10 transformation followed by paired *t*-test was used to compare mRNA levels in a-CD3/a-CD28-activated PBMC with or without fisetin. (**B**) PBMC were activated for 48 h in the indicated conditions and secreted cytokines (IFN-γ, IL-17A, and IL-4) present in the cell culture media were measured by ELISA. Means ± SD are shown and the paired *t*-test was used to compare values between fisetin and no fisetin (–) for a-CD3/a-CD28-activated PBMC.

Because both keratinocyte and immune cell activation are involved in the initiation and chronicity of psoriasis and other inflammatory skin diseases [62,63,64], we next examined the effect of fisetin on anti-CD3/anti-CD28-activated PBMC secretion of inflammatory mediators. Subsequently, the mRNA expression level of type-1 and type-17 pro-inflammatory cytokines (*IFNG* and *IL-17A*) were analyzed when PBMCs were pretreated with fisetin (10 μM) for 10 min followed by activation with anti-CD3/CD28 for 6 h. As shown in Figure 6A, pretreatment of PBMC with fisetin resulted in significant inhibition of *IFNG* and *IL17A* mRNA accumulation. Under these conditions, PBMC were cultured for 48 h and analyzed for the cytokine protein production and release by ELISA. Altogether, these results demonstrated a significant inhibitory effect of fisetin on IFN-γ and IL-17 secretion, in spite of no obvious effect on the type-2 cytokine IL-4 (Figure 6B), and thus inhibition of inflammatory cells induced responses associated with the modulation of the expression of critical pro-inflammatory cytokines in psoriasis.

### 3.6. Topical Application of Fisetin Modulates Psoriasis-Like Features, Suppresses Proliferation, and Modulates Differentiation in a T Cell-Induced Three-Dimensional (3D) Full-Thickness Reconstituted Human Skin Model of Psoriasis (FTRHSP)

**Figure 7:**
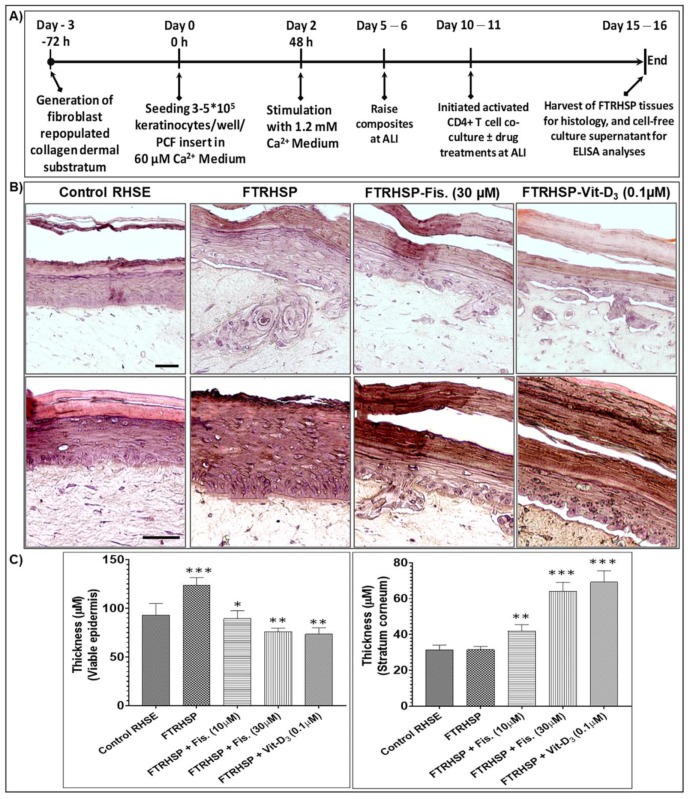
Fisetin modulated psoriasis-like features in a three-dimensional (3D) full-thickness reconstituted human skin model of psoriasis (FTRHSP). (**A**) Protocol for the establishment/generation of the 3D full-thickness reconstituted human skin model of psoriasis (FTRHSP), and for the evaluation of the therapeutic effects of compounds (fisetin or known control agent vitamin D_3_ (Vit-D_3_)) on psoriasis-like pathologic features. Candidate compounds were added on top of the tissue after five days of lifting to the air‒liquid interface and at the same day in wells containing preactivated or not T lymphocytes at the base of the engineered reconstructs and co-cultured. These were continually cultivated for an additional five days in the presence or absence of these agents prior to harvest, with media replenishment every alternate day. (**B**) Photomicrographs of modified Mayers’s hematoxylin- and eosin-stained control RHSE, FTRHSP, and FTRHSP treated with/without fisetin/Vit-D_3_ reconstructs, scale bar; top panel 20 μM, and bottom panel 50 µM. (**C**) Bar graphs showing quantification of changes in the thicknesses of; the viable epidermis (left panel), which significantly decreased in treated group vs. FTRHSP, and stratum corneum (right panel), which significantly increased in treated group vs. FTRHSP. The FTRHSP was generated and treated under different conditions (including with or without test agents fisetin or Vit-D_3_), and analyzed as detailed methods section. Significant differences between means ± standard deviation were determined as denoted by * p < 0.05, ** p < 0.01, and *** p < 0.001 vs. control RHSE.

Since human psoriatic skin lesion is phenotypically characterized by increases in epidermal hyperplasia, antimicrobial peptides, inflammation, and aberrant differentiation and signaling cascades [36,37,65,66], we determined the effect of fisetin on the established FTRHSP model, which mimics in vivo features of psoriatic skin lesions compared with Vit-D_3._ After five days of administering T cells to the 3D human skin equivalents (day 15 from start), tissues were harvested as above and molecular markers of epidermal differentiation, inflammation, and proliferation were determined as indicated in the protocol in Figure 7A. We observed that control FTRHSP (RHSE) tissues generated by co-culture with nonactivated CD4+ T cells showed more condensed epithelium. In contrast, the regenerated 3D FTRHSP, incorporating activated CD4+ T cells, recapitulated several features of human psoriatic lesion, with lesser cornification including increases in epidermal thickness, proliferation, and other changes in the aforementioned features (Figure 2B,C; data not shown). Furthermore, as previously reported [36,37], fisetin-treated FTRSHP tissues co-cultured with activated CD4+ T cells had a much thinner viable epidermis, similar to control tissues. These observations were comparable to the effects elicited by a single dose of Vit-D_3_ (0.1 µM) (Figure 7B,C).

**Figure 8:**
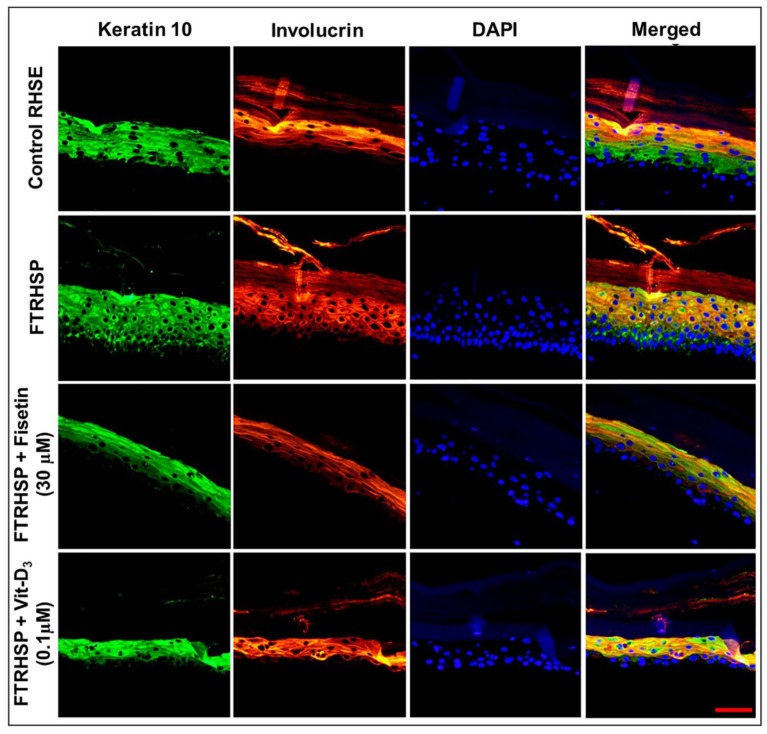
Fisetin modulates psoriasis-like features in the 3D full-thickness reconstituted human skin model of psoriasis (FTRHSP). Immunofluorescence staining was performed on FTRHSP sections by incubating with primary antibody against targets overnight at 4 °C followed by incubation with specific Alexa Flour 488-labeled secondary antibodies for 2 h at room temperature in the dark. Samples were mounted in mounting medium containing 4′,6-diamidino-2-phenylindole (DAPI) and analyzed microscopically. Immunofluorescence staining analysis of differential staining showing the protein expressions of early (K10) and late (involucrin) differentiation markers in control RHSE and FTRHSP under different treatment conditions. Stainings are shown in red and green, respectively, and DAPI in blue and results are representative images of two independent experiments each performed in quadruplicate in comparison to the control and treated FTRHSP. Scale bar = 20 μm.

**Figure 9:**
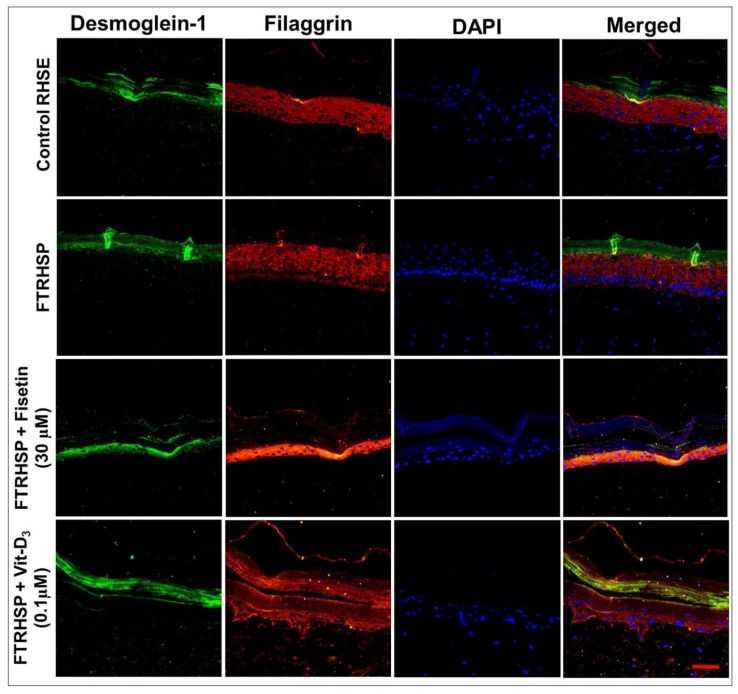
Representative immunofluorescent photomicrographs showing the protein expression levels of differentiation (filaggrin) and desmosomal (desmoglein-1) protein markers in control RHSE and FTRHSP under different treatment conditions versus fisetin or Vit-D_3_-treated FTRHSP tissues. Results are representative of three independent experiments each performed in quadruplicate and comparing control RHSE vs. FTRHSP treated groups. Data green (Dsgl-1); red (filaggrin), blue (DAPI) and mixed is merged representation. Dsgl-1 = desmoglein-1. Scale bar = 20 μm.

Additionally, by the analysis of immunostained FTRHSP tissues for the expression of psoriasis-related skin protein markers, we observed that, compared with control tissues, the terminal differentiation process was perturbed, as demonstrated by increased expression of Ki67 (proliferation (data not shown)) and aberrant increase and expression of differentiation markers (filaggrin, K-10, involucrin, TGase-1, and junctional protein desmoglein-1) (Figure 8 and Figure 9, Figure S2). Interestingly, we observed that the topical application of fisetin or a single dose of Vit-D_3_ after five days markedly suppressed proliferation, accompanied by normalization/induction of the expressions of differentiation markers including K-10, involucrin, filaggrin, TGase-1, and desmoglein 1 in the spinous via granular epidermal cell layers of the FTRHSP (Figure 8 and Figure 9, Figure S2).

### 3.7. Topical Application of Fisetin Suppresses mTOR Activation and Inflammation in a T Cell-Induced 3D FTRHSP Model

**Figure 10:**
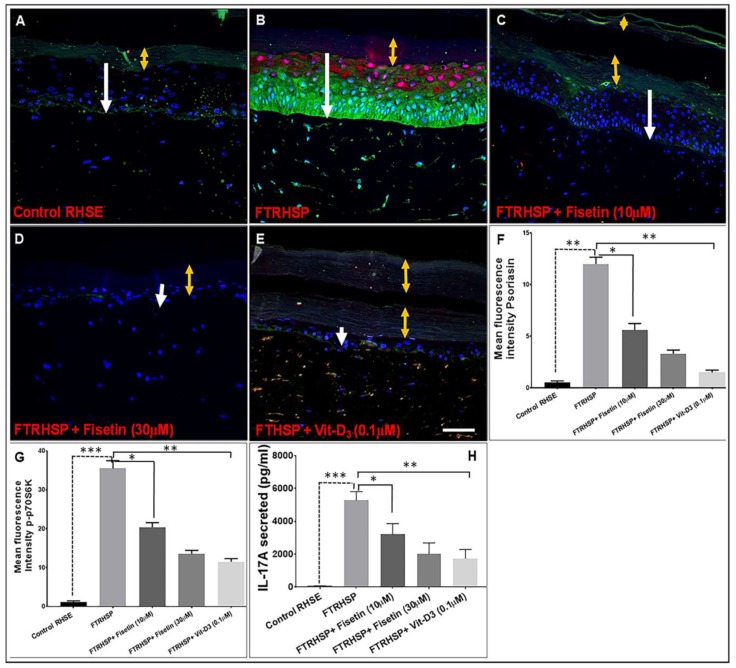
Fisetin modulated the expression of makers of psoriasis-like inflammation and mTOR activity in 3D human skin model of psoriasis. Immunofluorescence staining for p-p70S6K and Psoriasin was performed on FTRHSP sections by incubating with primary antibody against targets overnight at 4 °C followed by incubation with specific Alexa Flour 488-labeled secondary antibodies for 2 h at room temperature in the dark. Samples were mounted in mounting medium containing 4′,6-diamidino-2-phenylindole (DAPI) and analyzed microscopically. (**A**–**E**) the antimicrobial peptide (psoriasin) and mTOR activation effector, p-p70S6K stainings are shown in red and green, respectively, and DAPI in blue. Representative pictures are shown. Scale bar = 20 μm. The yellow arrows delineate the thickness of the stratum corneum, while the white arrows indicate the thickness of the viable epidermis. (**F**,**G**) For densitometric quantification, each color image was separated into its green, blue, and red channel components using ImageJ software (National Institutes of Health, Bethesda, MD, USA), and green and red channels were used to analyze mean fluorescent intensity. Data shown here are mean fluorescence intensity ± SEM. (**H**) Differential protein expression levels of IL-17A secreted in the conditioned media of FTRHSP cultures. Pro-inflammatory cytokines IL-17A in conditioned media, were analyzed when 3D FTRHSP were treated without and with fisetin (10–30 µM) treatment or 0.1 µM Vit-D_3_ for five days following activation by co-culturing with anti-CD3/CD28 activated CD4+ T cells for seven days as described in Section 2.12. Significant differences were determined and significance of comparisons were made for the expression levels of the target proteins of FTRHSP tissues versus fisetin or Vitamin D_3_-treated FTRHSP, corresponding to the solid lines, and also between nonactivated T cell-exposed control RHSE versus FTRHSP tissues, indicated by the broken lines, as denoted by * p < 0.05, ** p < 0.01, and *** p < 0.001.

Next, we examined the effect of fisetin on the mTOR pathway, the pro-inflammatory alarmin psoriasin (which acts as an antimicrobial peptide), and the secretion of pro-inflammatory cytokine IL-17A in the FTRHSP tissues and conditioned media, respectively, and compared it with Vit-D_3_ by employing immunofluorescence and ELISA analyses. Deregulated expression of these markers was associated with psoriatic skin lesions in the FRTHSP tissues and supernatants. We observed that, compared with control tissues, the activated T cells co-cultured 3D FTRHSP tissues showed increased staining/induction expression of psoriasin and p-p70S6K (Figure 10A–G), as well as increased secretion of IL-17A (Figure 10H). Topical application of fisetin or Vit-D_3_ suppressed the observed increases in the protein expression of psoriasin, p-p70S6K, and IL-17A in the established FTRHSP (Figure 10).

## 4. Discussion

In spite of recent progress in the understanding of the underlying molecular basis of psoriasis pathogenesis, existing treatment strategies still yield fluctuating and often elusive outcomes; hence, some effective treatments are generally associated with serious side effects. Also, recent efficacious synthetic agents are unrealistically expensive. Therefore, it is necessary to look into cheaper, safe, and cost-effective antipsoriatic agents. In this respect, medicinal plants have long been used as traditional remedies and are still used by over 75% of the world’s population to meet healthcare needs (World Health Organization, 2013 [67]. Bioactive natural ingredients hold promise and remain a source of potential new therapeutics, because the identification of their constituent bioactive phytochemicals allows for the development of novel, clinically useful, cheaper, and relatively safe drugs [68].

In this study, we demonstrated that fisetin has direct prodifferentiative, antiproliferative, and anti-inflammatory effects on both 2D and 3D cultures of keratinocytes and immune cells. To our knowledge, this is the first report indicating a direct effect in simplified and complex 3D systems of psoriasis-like disease. To investigate the effect of fisetin on NHEK differentiation, we selected differentiation markers relevant to psoriatic skin lesions, reflecting different stages of differentiation. K1 and K10, “often regarded as early keratinization markers [2],” are among the first proteins expressed by differentiating keratinocytes, indicative of the fundamental switch from the basal proliferative to the postmitotic phenotype. This switch is dysregulated in human and murine psoriatic skin lesions, and psoriatic keratinocytes maintain their proliferative capacity into the suprabasal and granular layers [4,6,10,30,38,51,69]. We report that fisetin significantly induced protein expression of the early, intermediate, and late markers of keratinocyte differentiation after 24–48 h of treatment. The biphasic effect of fisetin on K10 and other early markers, with concentrations greater than 10 µM inducing less expression levels of K10 and caspase-14, is likely because higher concentrations of fisetin drive keratinocytes to late differentiation. At later stages of differentiation, a decline in K10 and caspase-14 expression is expected, accompanied by a dose-dependent increase in TGase activity and protein expression, a late marker of differentiation that facilitates terminal differentiation and formation of the cornified envelope [48]. Thus, increased expression of K10 and TGase-1 delineates the effect of fisetin not only to initiate keratinocyte differentiation, but also to complete the barrier integrity stage. Furthermore, psoriatic lesions exhibit aberrant and reduced distribution and expression of epidermal TGase [70], and other differentiation markers including caspase-14, filaggrin, and involucrin, etc., resulting in hyperproliferation and parakeratotic skin [38,71,72]. Cutaneous inflammation in hyperplastic psoriasis skin lesions is often associated with increased expression of markers of proliferation, inflammation, and proinflammatory factors. The characteristic hallmarks of psoriasiform disease include the antimicrobial peptides psoriasin (S100A7) and koebnerisin (S100A15), two epidermal S100 calcium-binding proteins [66,73,74] with distinct roles but both serving as proinflammatory alarmins and chemoattractants [75], and cytokines. Moreover, the overexpression of the components of mTOR/PI3K/Akt and associated signaling has been associated with psoriatic disease [30,31,76]. In this study, fisetin (1‒40 µM) treatment of NHEKs significantly increased TGase enzyme activity by 13–120%, as well as the protein expressions of TGase-1 and other differentiation markers often observed to be downregulated in lesioned psoriatic skin [38,44]. Therefore, the antiproliferative and prodifferentiative effects of fisetin on keratinocytes suggest that fisetin may correct the proliferative and differentiation imbalances observed in psoriatic keratinocytes.

Importantly, the effect of fisetin was examined on inflammation, another major hallmark of psoriasis, by analyzing the expression and secretion of key psoriatic cytokines induced by known activators of human keratinocytes and immune cells. These immune-cell-derived cytokines are capable of activating keratinocytes, thereby exacerbating flare-ups [10]. Therefore, the role of inflammatory and type-17 cytokines in the psoriatic cytokine network has led to the development of antipsoriatic biologic drugs targeting TNF-*α* and IL-17A. Examples include etanercept (Enbrel^®^), a TNF-α antagonist and secukinumab, an antibody anti-IL-17A antagonist for psoriasis treatment [77,78,79]. Furthermore, high serum levels of TNF*α* and IL-6 are observed in psoriasis patients compared with healthy controls [80], and IL-1*α*, which is primarily produced by skin keratinocytes, regulates proliferation and differentiation and dictates immune function [81], via priming IL-23-induced modulation of IL-17A production by T cells [82,83,84,85]. Overexpression of IL-1α in basal epidermal layers of transgenic mouse model leads to a psoriasis-like phenotype with keratinocyte hyperplasia and immune cell infiltration [86,87,88]. Here, inflammatory cytokine production in keratinocytes was stimulated by a known inducer of keratinocyte inflammatory response, TPA [61], while PBMCs were activated by anti-CD3/anti-CD28 treatment, which mimics antigen presentation to lymphocytes [40]. Since keratinocytes are the major source of a spectrum of different cytokines including IL-1, IL-6, and TNFα in skin inflammatory responses [8,10,89], by stimulating NHEKs with TPA, we presumably mimicked the initiation phase of the psoriatic process [38,90]. Our data on both multiplexed and sandwich ELISAs showed that fisetin was capable of efficiently inhibiting the production of inflammatory cytokine induced in keratinocytes, PBMCs, and activated-CD4+ T cells co-cultured with 3D FTRHSP. Among the cytokines downregulated by fisetin were Th-1/Th-17 cytokines, including IL-17A and IFN-γ. Therefore, we propose that fisetin, by inhibiting proliferation, inducing differentiation, and downregulating the psoriatic cytokine network in keratinocytes and immune cells, could restore keratinocyte and skin homeostasis, and may act as a possible antipsoriatic agent, as suggested by the 3D psoriatic skin model studies. Furthermore, these findings suggest that fisetin exerts inhibitory functions on the two major cell types involved in psoriasis pathogenesis, keratinocytes, and immune cells by inhibiting the activation of PI3K/AKT/mTOR and MAPK pathway components. The induction of ERK1/2 activation by fisetin is a mechanistic phenomenon observed in several other studies by our team and others. This is reflected in relation to the activation of cutaneous keratinocyte differentiation (specialized program cell death in keratinocytes) rather than the expected induction of proliferation [91,92], and the result is not surprising as constitutive activation of ERK is related to apoptosis. Targeting immune cells and their mediators is the ultimate approach to treat psoriasis. Moreover, considering the essential role of keratinocyte crosstalk with immune cells in inflammatory skin diseases like psoriasis, targeting the inflammatory mediator network from both keratinocytes and immune cells, as well as the antiproliferative and prodifferentiation potential, may enhance the antipsoriatic effect of a compound such as fisetin. Considering that fisetin exerts a pleiotropic effect, targeting the three major hallmarks of psoriasis as a single, natural agent, and is safe and effective, fisetin may also be less compromising to the patient’s immune system. The observed increased expression of psoriasin (S100A7), an antimicrobial peptide in the generated CD4+ T cells activated in 3D FTRHSP compared to normal FTRHSP, was suppressed by topical fisetin treatment to values comparable to the suppression induced by physiologic Vit-D_3_ [60,75,93]. We showed earlier the increased expression of keratinocyte-induced immune mediator secretion without immune-cell-derived cytokines in psoriatic skin equivalents [37], which are critical facets in the pathogenesis of psoriasis [93]. Herein, we observed that fisetin inhibited the increased release of both keratinocyte and activated immune-cell-associated Th1/Th-17 pro-inflammatory cytokines production, including IL-17A, TNF-a, IFN-γ, etc. It is important to note that the inculcated keratinocytes and activated immune cells in the currently established FTRHSP model elicit and mimic the feedback loop often induced by specific immune cells in the immunopathogenesis of psoriatic skin lesions. In view of the intricacy of psoriasis etiology, it is noteworthy that agents like fisetin that are capable of rescinding numerous critical endpoints in psoriasiform disease are prospective therapeutic agents for the control of psoriasis.

To the best of our knowledge, this study demonstrates for the first time the direct effects of fisetin on psoriasis-like features in 2D and 3D psoriasis-like systems, and thus highlights its likely multifactorial prospect as an agent from natural botanical sources consumed on a daily basis. This could pique interest in its development for preventing or slowing the progression of psoriasis and other inflammatory diseases. Furthermore, granted that the current model is promising, additional studies employing complementary animal and humanized murine psoriasis-like skin models that integrate more of the immune axis (e.g., activated dendritic cells) are needed to further corroborate the detailed mode of action and efficacy of fisetin without involving too much animal experimentation. Taken together, the current study provides a rationale for future preclinical proof-of-concept studies leading to clinical studies to evaluate fisetin for the control of psoriasis in human patients.

## Supplementary Materials

The following are available online at https://www.mdpi.com/2073-4409/8/9/1089/s1, Figures S1 and S2: Figure S1: Fisetin significantly and dose-dependently suppressed the IL-22 induced proliferation of 2D culture NHEKs by MTT assay, compared with precancerous (HaCaT) and cancer (A431) cell lines. Figure S2: Representative photomicrographs of immunofluorescent staining showing the protein expression levels of differentiation marker (TGase-1) in control (RHSE) and FTRHSP conditions versus (fisetin or vit-D3)-treated FTRHSP tissues. Data green (TGase-1); blue (DAPI) and mixed staining is merged representation. Results are representative of three independent experiments each performed in quadruplicate and comparing control RHSE vs treated FTRHSP. TGase-1 = transglutaminase-1. Scale Bar = 20 µm.
