# Supplementary material for: Fisetin, a 3,7,3′,4′-Tetrahydroxyflavone Inhibits the PI3K/Akt/mTOR and MAPK Pathways and Ameliorates Psoriasis Pathology in 2D and 3D Organotypic Human Inflammatory Skin Models
Source: Cells. 2019 Sep 15;8(9):1089. doi: 10.3390/cells8091089 (PMC6770767; doi:10.3390/cells8091089)
Supplement: Supplementary file 1 [file cells-08-01089-s001.pdf]

Article

Fisetin, a 3, 7, 3′, 4′-tetrahydroxyflavone inhibits PI3K/Akt/mTOR and MAPK Pathways and Ameliorates Psoriasis Pathology in 2D and 3D Organotypic Human Inflammatory Skin Models

Jean Christopher Chamcheu[1,*], Stephane Esnault[2], Vaqar M. Adhami[3], Andrea L. Noll[2], Sergette Banang-Mbeumi[1,#], Tithi Roy[1], Sitanshu S. Singh[1], Shile Huang[4,5], Konstantin Gus Kousoulas[6], and Hasan Mukhtar[2]

Supplementary Materials:

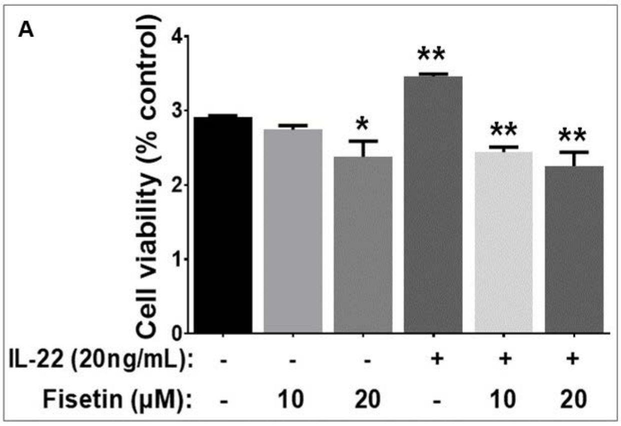

Figure S1: Fisetin significantly and dose-dependently suppressed the IL-22 induced proliferation of 2D culture NHEKs by MTT assay, compared with precancerous (HaCaT) and cancer (A431) cell lines *(**p<0.05 to ***p<0.001)*.

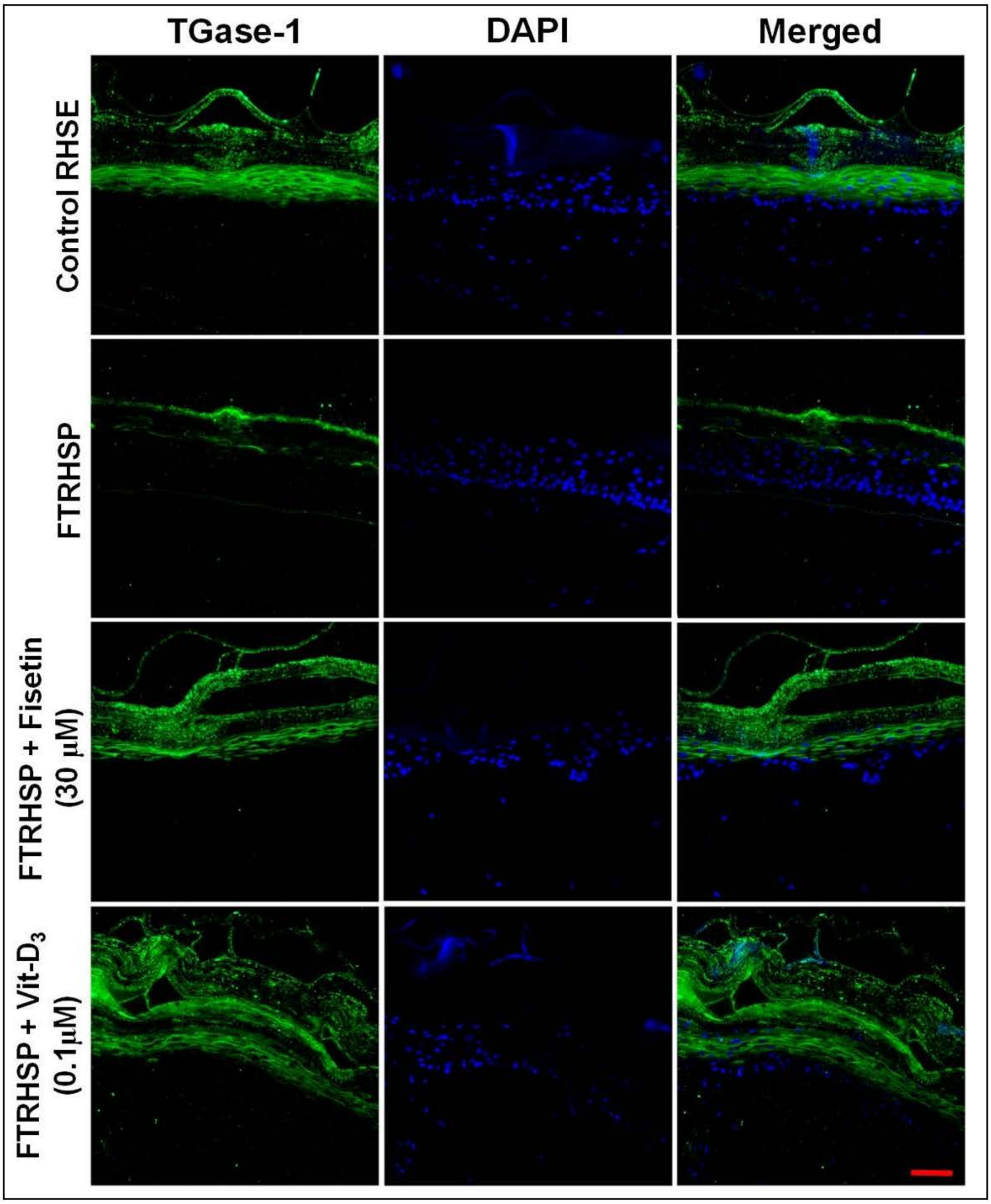

Figure S2: Representative photomicrographs of immunofluorescent staining showing the protein expression levels of differentiation marker (TGase-1) in control (RHSE) and FTRHSP conditions versus (fisetin or vit-D_3)-treated FTRHSP tissues. Data green (TGase-1); blue (DAPI) and mixed staining is merged representation. Results are representative of three independent experiments each performed in quadruplicate and comparing control RHSE vs treated FTRHSP. TGase-1 = transglutaminase-1. Scale Bar = 20 μm.
